# The genomic landscape of mammal domestication might be orchestrated by selected transcription factors regulating brain and craniofacial development

**DOI:** 10.1007/s00427-023-00709-7

**Published:** 2023-08-08

**Authors:** Antonio Benítez-Burraco, Juan Uriagereka, Serge Nataf

**Affiliations:** 1 Department of Spanish, Linguistics, and Theory of Literature (Linguistics), Faculty of Philology, University of Seville, Seville, Spain; 2 Área de Lingüística General, Departamento de Lengua Española, Lingüística y Teoría de la Literatura, Facultad de Filología, Universidad de Sevilla, C/ Palos de la Frontera s/n., 41007-, Sevilla, España; 3 Department of Linguistics and School of Languages, Literatures & Cultures, University of Maryland, College Park, MD USA; 4 Stem-cell and Brain Research Institute, 18 avenue de Doyen Lépine, F-69500 Bron, France; 5 University of Lyon 1, 43 Bd du 11 Novembre 1918, F-69100 Villeurbanne, France; 6 Bank of Tissues and Cells, Hospices Civils de Lyon, Hôpital Edouard Herriot, Place d’Arsonval, F-69003 Lyon, France

**Keywords:** Domestication, Transcription factors, Candidate genes, Brain, Craniofacial region, SOX2

## Abstract

Domestication transforms once wild animals into tamed animals that can be then exploited by humans. The process entails modifications in the body, cognition, and behavior that are essentially driven by differences in gene expression patterns. Although genetic and epigenetic mechanisms were shown to underlie such differences, less is known about the role exerted by trans-regulatory molecules, notably transcription factors (TFs) in domestication. In this paper, we conducted extensive in silico analyses aimed to clarify the TF landscape of mammal domestication. We first searched the literature, so as to establish a large list of genes selected with domestication in mammals. From this list, we selected genes experimentally demonstrated to exhibit TF functions. We also considered TFs displaying a statistically significant number of targets among the entire list of (domestication) selected genes. This workflow allowed us to identify 5 candidate TFs (SOX2, KLF4, MITF, NR3C1, NR3C2) that were further assessed in terms of biochemical and functional properties. We found that such TFs-of-interest related to mammal domestication are all significantly involved in the development of the brain and the craniofacial region, as well as the immune response and lipid metabolism. A ranking strategy, essentially based on a survey of protein-protein interactions datasets, allowed us to identify *SOX2* as the main candidate TF involved in domestication-associated evolutionary changes. These findings should help to clarify the molecular mechanics of domestication and are of interest for future studies aimed to understand the behavioral and cognitive changes associated to domestication.

**Supplementary Information:**

The online version contains supplementary material available at 10.1007/s00427-023-00709-7.

## Introduction

Domestication is the process that transforms once wild animals into tamed animals after extensive contact with humans. Understanding domestication is crucial for diverse and productive domestic animal varieties, but also for testing how evolution works, particularly in response to quick changes in social environment. Trends towards increased prosocial behavior, without human intervention, have been claimed to trigger domesticated features also in wild species, as observed in bonobos (Hari et al. 2012) and hypothesized for humans (Hare 2017), but also in cases of commensalism (Brooker et al. 2020).

Domestication impacts on the body, cognition, and behavior of animals, with relevant changes becoming ultimately fixed and transmitted to the offspring. Being such a rapid process, domestication has been argued to result mostly from epigenetic changes (Trut et al. 2009a; Janowitz Koch et al. 2016; Vogt 2017; Vogt 2021). But when selective forces persist, epigenetic changes can be assimilated as genetic variants (O’Dea et al. 2016; Vogt 2021). Genetic differences between domesticated animals and wild conspecifics have been found for several species: the pig (Larson et al. 2005), the dog (Axelsson et al. 2013; Freedman et al. 2016), the cat (Montague et al. 2014), cattle (Qanbari et al. 2014), the rabbit (Carneiro et al. 2014), or the horse (Pendleton et al. 2018). Overall, domestication seems to result from subtle changes in multiple regulatory networks and, ultimately, in many genes, each finely contributing to the phenotype. There is only partial overlap across species between candidate genes (see Wilkins et al. 2014a for review); still, animals that have been domesticated for millennia share a set of distinctive features, commonly referred to as the *domestication syndrome*. This includes smaller skulls/brains, reduced teeth and snouts, neotenic features, earlier sexual maturation, altered hairiness and pigmentation, and reduced sexual dimorphism (Wilkins et al. 2014b). The features encompassing the syndrome are expressed variably in different species, with some occasionally absent (Sánchez-Villagra et al. 2016). It has been hypothesized that the co-occurrence of (some of) these traits stems from tameness resulting in socialization-induced molecular cues. Mostly related to the hypothalamic-pituitary-adrenal axis and ultimately associating to fear control and bounding, these cues alter the migration and fate of neural crest (NC) cells. These are a type of stem cells that contribute to the formation of body organs during embryonic growth (Wilkins et al. 2014a; Wilkins 2017). However, such a hypothesis and the very existence of a domestication syndrome have been debated (Sánchez-Villagra et al. 2016; Lord et al. 2020, and Johnsson et al. 2021). Indeed, domestication has induced physiological changes that vary greatly, depending on species and domestication contexts. Moreover, multiple phenotypic traits presently observed in domesticated mammals have been selected by humans for exploitation purposes, so they cannot be considered as resulting from socialization (i.e., selection for tameness) as such. Finally, the multifactorial nature of domestication is in line with most tame species having been domesticated at various places and historical times: present-day domesticated populations spring from an admixture of diverse domesticated subpopulations (Bruford et al. 2003; Larson et al. 2005, 2010). It thus appears particularly challenging to decipher the evolutionary mechanisms that were genuinely triggered by socialization in domesticated animals.

Most differences between domesticated and wild animals are thought to result from differences in gene expression patterns. Interestingly, (epi)mutations in the regulatory regions of key developmental genes have been shown to result in profound dissimilarities between domesticates and wild counterparts (see, e.g., Lindblad-Toh et al. (2005) on dogs vs. wolves). Studies have also uncovered changes in the methylation profiles of multiple genes between domestic and wild populations (e.g., Sundman et al. (2020) on dogs; Nätt et al. (2012) on chickens; or Albert et al. (2012a) on guinea pigs, pigs, and rabbits). Less is known about the trans-regulatory molecules, notably transcription factors (TFs), which orchestrate domestication-driven genomic events. Most studies have focused on the role of given TFs on particular traits that can be found selected in specific domesticated species (e.g., Baranowska Körberg et al. (2014) on the role of MITF-M on pigmentation changes in dogs). Identifying such TFs is important, as epigenetic modifications found to be transmissible (notably methylation of histone or DNA cytosines) impact the regulatory functions of TFs (Hughes and Lambert 2017; Yin et al. 2017).

In this paper, we explore the TF landscape of domestication, to consider the potential effect of TF activity changes on the phenotypic expression of domestication in mammals. We have uncovered a limited set of TFs potentially orchestrating the multiple genomic programs underlying domestication events in mammals. To identify such core TFs, we first searched the literature, so as to establish a large list of 764 genes selected with domestication in mammals. This list was then filtered out to retain TF genes. We also searched for TFs displaying a statistically significant number of targets among the whole list of domestication selected genes. This workflow allowed us to identify 5 candidate core TFs that were further assessed in terms of protein-protein interactions and functional properties. We found evidence indicating that the pathways and biological processes regulated by such TFs-of-interest are significantly involved in the development of both brain and craniofacial features—traits that are notably impacted by domestication events. Potential consequences for the emergence of the domesticated phenotype in mammals are subsequently discussed. Although the set of TFs we highlight in the paper opens a promising window into the regulatory aspects of mammal domestication, other contributors such as microRNAs and other non-coding RNAs are likely involved and deserve a closer examination in future studies.

## Materials and methods

**Fig. 1:**
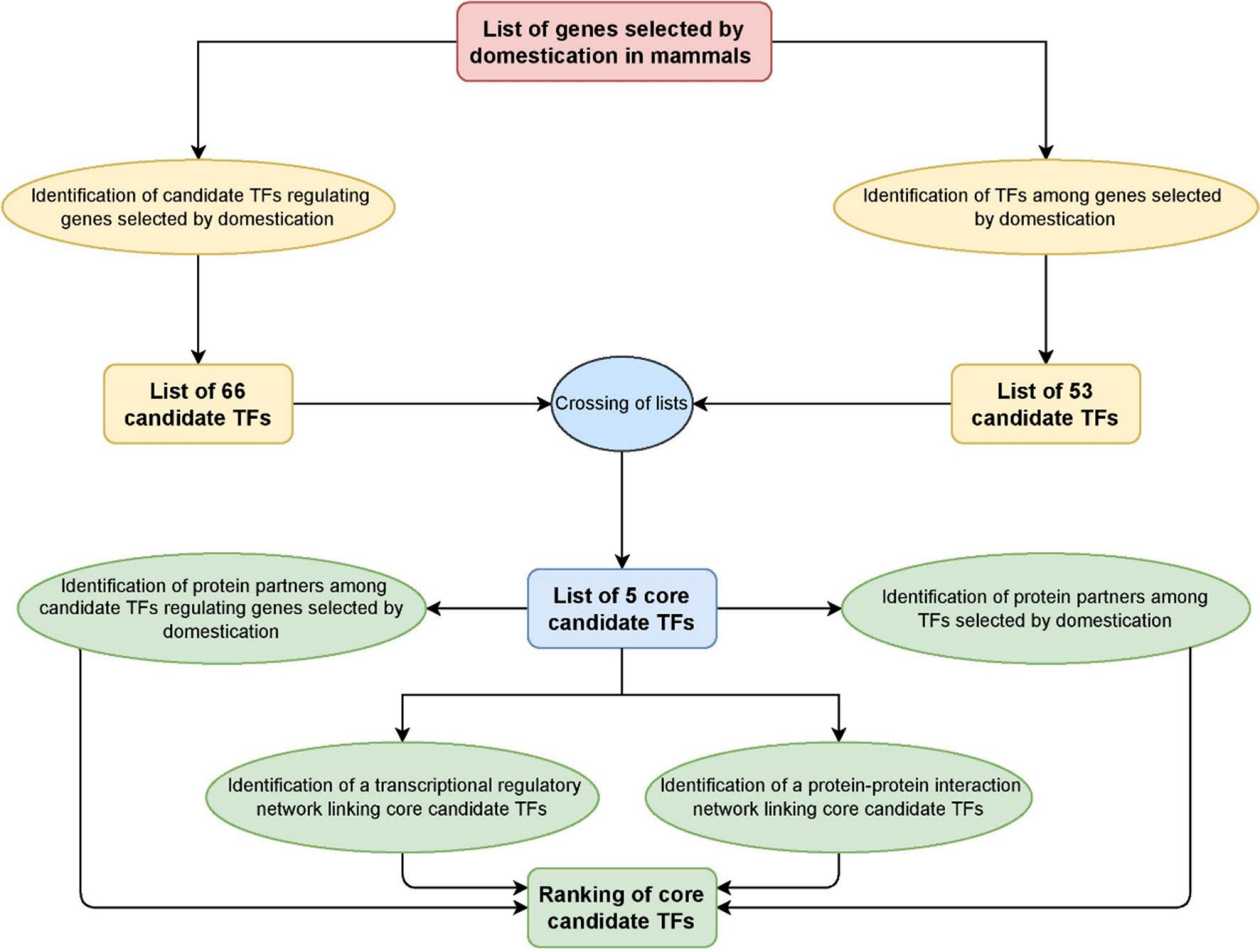
Analytical workflow. The main analytical tasks (in oval shapes) and resulting data (in rectangular shapes) are grouped by colors indicating successive chronological steps of the workflow

We first compiled a list of genes that show signals of selection in domesticated mammals, compared to their non-domesticated counterparts. For this, we consulted general bibliographic databases like PubMed (https://pubmed.ncbi.nlm.nih.gov/) and Google Scholar (https://scholar.google.com/) with “selection “+ “genes” + “domestication” as search criteria. We then selected papers focusing on mammal domesticated species vs. their wild counterparts. The species we relied on include guinea pig, pig, rat, dog, cat, cattle, domesticated fox, horse, rabbit, and sheep (Womack 2005; Trut et al. 2009b; Albert et al. 2012b; Axelsson et al. 2013; Bellone et al. 2013; Carneiro et al. 2014; Montague et al. 2014; Qanbari et al. 2014; Schubert et al. 2014; Wilkins et al. 2014b; Wright 2015; Cagan and Blass 2016; Freedman et al. 2016; Zapata et al. 2016; Benítez-Burraco et al. 2017; Theofanopoulou et al. 2017; Pendleton et al. 2018). The list includes 764 genes (Supplemental Data File 1; sheet 1). The enrichment analysis platform Enrichr (Kuleshov et al. 2016; Xie et al. 2021) was used to perform enrichment analyses in pathways and TF targets. Briefly, pathway enrichments with an adjusted *p* value < 0.01 were extracted from the analyses of 4 libraries: Reactome 2022 (Gillespie et al. 2022), Bioplanet 2019 (Huang et al. 2019), KEGG 2021 (Kyoto encyclopedia of genes and genomes) (Kanehisa et al. 2021), and Martens et al. 2021 (Martens et al. 2021). These libraries gather lists of genes that, based on the available literature, can be regarded as involved in specific regulatory pathways. Naturally, these 4 libraries may exhibit differences concerning the number and nature of pathways to which a gene list can be associated. To fix this issue, we combined the outcomes of the survey of each individual library then extracting and ranking the most significant enrichments across the 4 libraries. The principle underlying pathway enrichment analyses can be summarized as follows. Considering the existence of roughly 22,000 protein-coding genes in the human or murine genome, one can determine if a given set of genes harbors a higher-than-expected number of genes involved in one specific pathway. Then, for each enrichment found, a *p* value is calculated, generally based on the Fisher exact test. For our analyses, we used the Enrichr platform (https://maayanlab.cloud/Enrichr/), which provides a computed adjusted *p* value based on a corrected Fisher exact test (see Kuleshov et al. 2016 for details). For enrichment analyses in TF targets, we queried the ChEA 2022 library of experimentally demonstrated TF targets and binding sites, which is mostly based on ChIP-seq results. The principle underlying TF targets enrichment analysis can be summarized as follows. ChEA 2022 is a library of ChIP-seq data consisting of sets of genes that have been experimentally shown to be regulated by specific TFs. Each TF is thus associated to a specific set of experimentally demonstrated target genes out of a total of roughly 22,000 human protein-coding genes in the human or murine genome. Accordingly, one can determine if a given list of genes harbors a higher-than-expected number of genes previously verified to be targeted by a given TF. Finally, we used the Harmonizome website (https://maayanlab.cloud/Harmonizome/), an Enrichr-connected integrated resource of OMICs datasets (see Rouillard et al. (2016) for details), to obtain the lists of experimentally demonstrated targets of each of our candidate TFs and to perform pathway enrichment analyses on these lists, following the same strategy described above. Data mining analyses were performed in triplicate between October 2022 and January 2023. The general workflow applied to this study is summarized in Fig. 1.

## Results

We first considered whether genes selected in domesticated mammals significantly comprise genes coding for TFs. By crossing the list of domestication selected genes with currently known human TFs (Lambert et al. 2018) (Supplemental Data File 2; sheet 1), we identified 53 TFs which are thus candidate transcriptional regulators of domestication processes (Table 1; left) (Supplemental Data File 2; sheet 1). Generally, the set was significantly enriched in TFs involved in pathways which are poorly specific (such as “gene expression” or “cell differentiation”) (Supplemental Data File 2; sheet 2). Among the most statistically significant pathways, we found “neural crest differentiation” (adjusted *p* value: 1.60E^−5^) and “oligodendrocyte specification and differentiation, leading to myelin components for CNS” (adjusted *p* value: 4.54E^−5^), as well as pathways involved in beta-catenin signaling (“deactivation of beta-catenin transactivating complex”, adjusted *p* value: 0.004) and Wnt signaling (“Wnt signaling pathway”, adjusted *p* value: 0.006) (Supplemental Data File 2; sheet 2).

**Table 1:** TFs potentially involved in mammal domestication

TFs subject to a positive selection process in domesticated mammals	TFs targeting a statistically significant (adjusted *p* value < 0.01) number of genes positively selected in domesticated mammals
ARID3B	AF4
CBX2	AR
CUX2	ARNT
DMRT3	BRD4
EEA1	CDX2
ELF2	CEBPA
ETV4	CEBPD
FOXD3	CTBP1
FOXI1	CTBP2
FOXJ3	CTCF
GRHL3	CTNNB1
HMGA2	DROSHA
IKZF1	FLI1
JRKL	FOXA1
KLF4	FOXA2
LIN28B	FOXM1
LTF	GATA1
MAFK	GATA2
MBD2	GF1
MITF	GF1B
NPAS3	JARID2
NR2F2	KDM2B
NR3C1	KLF1
NR3C2	KLF4
NRF1	KLF5
OLIG1	LEF1
PAX2	LMO2
PAX3	LUZP1
PHF20	MBD3
PLAG1	MEIS1
PPARD	MITF
PRMT3	MTF2
SETBP1	NFKB1
SKI	NR3C1
SOX10	NR3C2
SOX2	OCT4
SOX6	OLIG2
SOX9	P300
SREBF1	POU3F2
TFCP2L1	POU5F1
THYN1	PPAR
TLX3	REST
ZFAT	RING1B
ZNF236	RUNX2
ZNF286A	SMAD3
ZNF286B	SMAD4
ZNF436	SMARCA4
ZNF492	SMARCD1
ZNF516	SOX11
ZNF521	SOX2
ZNF555	STAT1
ZNF679	STAT3
ZNF780B	SUZ12
	TAL1
	TBX3
	TCF3
	TCF4
	TEAD4
	TOP2B
	TP53
	TP63
	UBTF
	WT1
	YAP1
	ZFP57
	ZNF217

As a complementary approach, we determined whether the list of genes selected with domestication was enriched in genes previously found to be regulated by specific TFs. To this aim, we surveyed the data library “ChEA 2022”, which gathers results from 757 ChiP-seq (or ChIP-seq-related) experiments performed in a large variety of human or rodent cell types. By this method, we identified 66 TFs exhibiting among their targets a statistically significant number of genes selected with domestication (adjusted *p* value < 0.01) (Table 1, right column) (Supplemental Data File 3; sheets 1 and 2). Again, this list was significantly enriched in TFs involved in poorly specific pathways such as “pre-implantation embryo”, “signaling pathways regulating pluripotency of stem cells”, or “mesodermal commitment pathway” (Supplemental Data File 3; sheet 3). However, a significant enrichment was also observed for specific pathways of interest for the domesticated phenotype, notably including “TGF-beta Receptor Signaling” (adjusted *p* value: 4.97E^−10^), and again beta-catenin signaling “Nuclear beta-catenin signaling and target gene transcription regulation” (adjusted *p* value: 2.10E^−8^), and Wnt signaling “Wnt signaling pathway” (adjusted *p* value: 2.01E^−7^). It is noteworthy that a significant enrichment was also found for TFs involved in the transcriptional regulation by RUNX1 (adjusted *p* value: 1.25E^−5^), RUNX2 (adjusted *p* value: 6.94E^−4^), and RUNX3 (adjusted *p* value: 1.23E^−6^) (Supplemental Data File 3; sheet 3).

It should be underscored that the sets of pathways found to be enriched for TFs’ list 1 (*n* = 53) and list 2 (*n* =66) did not overlap, except for the beta-catenin signaling pathway. For this reason and because our goal was to establish a set of candidate master TFs regulating a putative domestication-associated transcriptional program, we crossed our two lists of candidate TFs. This enabled us to identify TFs that were selected in domesticated mammals and that regulate a statistically significant number of genes selected during domestication. We found five overlapping TFs: KLF4, MITF, NR3C1, NR3C2, and SOX2. We, thus, concluded that these five TFs represent the main candidate TFs regulating the transcriptional landscape of domestication in mammals. Table 2 contains some functional features of interest within these TFs.

**Table 2:** Functional characterization of core TFs involved in mammal domestication

KLF4	- Involved in cell growth, proliferation, and differentiation, including the induction of pluripotent stem cells (Ghaleb and Yang 2017)
MITF	- Involved in NC-derived melanocyte development and differentiation (Hershey and Fisher 2005) - Mutations of the gene result in auditory-pigmentary syndromes categorized as neurocrestopathies (e.g., Waardenburg syndrome) (Hershey and Fisher 2005)
NR3C1	- Encodes the glucocorticoid receptor (GR), with glucocorticoid levels affecting stability of dendritic spines (Bennett and Lagopoulos 2014) - Undergoes epigenetic modifications in response to in utero stress (Duffy et al. 2019), epigenetic modifications associated to stress and fear regulation being an aspect of domestication (Jensen 2015)
NR3C2	- Encodes the aldosterone or mineralocorticoid receptor (MR), which also binds glucocorticoids, involved in sodium reabsorption and potassium excretion (Le et al. 2004; Baker and Katsu 2017) - Candidate for autism spectrum disorders (Turner et al. 2016)
SOX2	- Encodes one component of the SHH-GLI signaling pathway, which regulates the fate of NC cells (Oosterveen et al. 2012; Oosterveen et al. 2013; Peterson et al. 2012) - Contributes to the maintenance of cell totipotency during embryonic development, the pluripotency of embryonic stem cells, and the multipotency of neural stem cells (Sarlak and Vincent 2016) - Plays key role in neurogenesis, and neuronal and glial differentiation of NC-derived cells (Wakamatsu and Uchikawa 2021; Mercurio et al. 2022) - Also involved in adult tissue homeostasis, particularly in the central nervous system (Feng and Wen 2015), contributing to the development of specific brain areas, like the ventral telencephalon (Ferri et al. 2013) or the hippocampus (Mercurio et al. 2021) - Also involved in the formation of the sensorimotor system, including the eye, the ear, and the pituitary (Kondoh et al. 2004; Kelberman et al. 2008; Dvorakova et al. 2020), as well as its connectivity with the cortex (Mercurio et al. 2019) - Contributes to tooth development via Wnt signaling (Lee et al. 2016) - Mutations in the gene result in eye abnormal growth, brain malformations (particularly impacting the hippocampus and the forebrain), developmental delay (including intellectual disability and growth delay), and abnormal gonadal growth (Hever et al. 2006; Williamson et al. 2006; Tziaferi et al. 2008; Dash et al. 2020; Mercurio et al. 2022)

We then aimed at determining how the functional roles currently assigned to these core candidate TFs may relate with domestication-associated events. To achieve this goal, we first performed enrichment analyses on the lists of the known targets of 4 of our core candidates, as experimentally demonstrated by ChIP-seq (or ChIP-seq-related) experiments (no data were available for NR3C2 via the ChEA 2023 library) (Supplemental Data File 4; sheet 1 to 4). As shown in Table 3, among the only 6 pathways that are enriched across the lists of TF targets, 2 are directly related to the brain (namely, “axon guidance” and “BDNF signaling pathway”, adjusted *p* values < 0.01) (Supplemental Data File 4; sheets 1 to 4).

**Table 3:** Shared pathway enrichments across lists of genes targeted by SOX2, KLF4, MITF, and NR3C1

Signal transduction	
Axon guidance	
BDNF signaling pathway	
Interleukin-2 signaling pathway	
TGF-beta regulation of extracellular matrix	
VEGFA-VEGFR2 signaling pathway	
Adipogenesis	

**Fig. 2:**
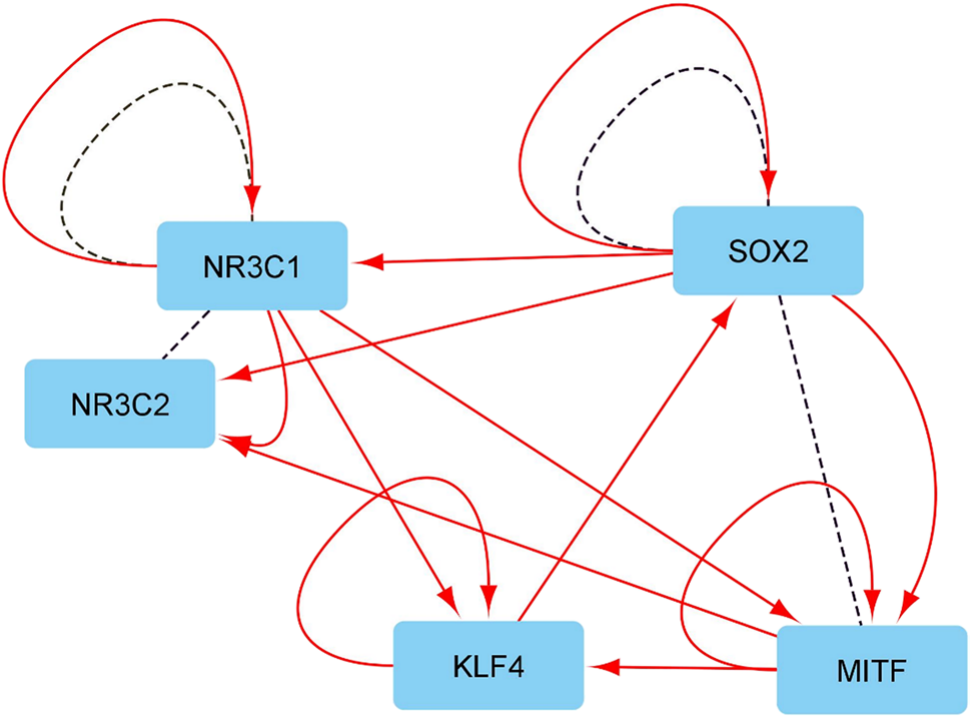
Regulatory and interaction network linking core TFs involved in mammal domestication. Red plain arrows indicate transcriptional regulatory links. Black dashed lines indicate protein/protein interactions

We then sought to identify a potential key regulatory TF among our short list of five candidate TFs. To this aim, we first surveyed the ChEA 2022 databank extracting transcriptional regulatory links between our five TFs (Fig. 1). In the identified regularity network linking these five TFs, SOX2 exhibited the highest number of targets among candidate TFs. Additionally, we surveyed the proteomics databank “BioGrid” to find out about potential interactions between these five core candidates together with the list of 53 TFs subject to a positive selection process in domesticated mammals (Table 4, central column) and the list of 66 TFs targeting a significant number of genes positively selected in domesticated mammals (Table 4, right column) (Supplemental Data File 5; sheets 1 to 5). We found that SOX2 exhibits by far the highest number of partners among both lists (Table 4, Fig. 2, and Supplemental Data File 5; sheet 1 to 5).

**Table 4:** TF protein partners of core TFs involved in mammal domestication

Core candidate TFs	Protein partners of core candidate TFs *Among domestication selected TFs*	Protein partners of core candidate TFs *Among TFs targeting a significant number of domestication selected genes*
SOX2	ARID3B CBX2 ELF2 MBD2 MITF SOX2 SOX6	ARNT CEBPD CTBP1 CTBP2 CTCF CTNNB1 FLI1 KDM2B LUZP1 MBD3 MITF POU5F1 RUNX2 SMARCA4 SMARCD1 SOX2 TBX3 TCF3 TP63 UBTF YAP1
MITF	SOX2	SOX2 LEF1
KLF4	ZNF516	AR KDM2B
NR3C1	ARID3B FOXJ3 NR2F2 NR3C1 NR3C2 ZNF516	CEBPA KDM2B NFKB1 NR3C1 NR3C2 SMAD3 STAT3 TP53
NR3C2	NR3C1	NR3C1 TP53

## Discussion

We have delved into the regulatory landscape of mammal domestication via different in silico analyses, aimed at identifying TFs potentially contributing to domestication features, as well as core molecular pathways significantly targeted by these TFs. We found that several pathways stand out as particularly relevant, in light of previously published studies: the pathway depending on RUNX2, the Wnt/β-catenin signaling pathway, and the neural crest differentiation pathway. *RUNX2* is one osteogenic master gene involved in skull morphogenesis (Lattanzi 2016), but also in brain development, particularly thalamic and hippocampal GABAergic neurons (Pleasure et al. 2000; Benes et al. 2007; Reale et al. 2013). RUNX2 deficits result in cleidocranial dysplasia, a condition involving reduced skull ossification; RUNX2 overexpression is associated to syndromic craniosynostosis (Lattanzi 2016). RUNX2 has been related to morphological variation in dog breeds, particularly with differences in limb and skull morphology, the latter being a universal target of domestication (Fondon 3rd and Garner 2004). In our analysis, we have found that RUNX2 targets a significant number of genes positively selected in domesticated mammals. Additionally, our findings give some support to the NC hypothesis of domestication (Wilkins et al. 2014a; Wilkins 2017), as we found a significant enrichment in TFs involved in NC differentiation and Wnt/β-catenin signaling, a molecular pathway playing a key role in NC cell induction and differentiation (Wu et al. 2003; Hari et al. 2012; Leung et al. 2016).

Because the Runx2 pathway and the NC-related pathways—although partially overlapping—are distinct in nature, we tried to identify specific candidate TFs for domestication by searching for TFs sharing two features: (i) being selected in domesticated mammals and (ii) regulating a statistically significant number of genes selected with domestication. This resulted in a small set of TFs (SOX2, MITF, KLF4, NR3C1, NR3C2), with SOX2 exhibiting several functional and biochemical properties that may confer it a major role in domestication. Although SOX2 is known to be particularly important for brain development (Feng and Wen 2015; Wakamatsu and Uchikawa 2021; Mercurio et al. 2022), its specific role in domestication processes is not well known, despite its use as a generator of induced pluripotent stem cells (iPSCs) in large domesticated animals (Bressan et al. 2020). Nonetheless, as reflected in Table 2, SOX2 contributes to the development of most body parts impacted by domestication, like the teeth and brain areas known to be modified in domesticated mammals, such as the hippocampus (Mercurio et al. 2021). From our analyses, one can further conclude that, although SOX2 has not been selected in most domesticated species, the functions it contribute to may have been impacted by domestication, via selection of (i) TFs coding for SOX2 partners, (ii) genes targeted by SOX2, and/or (iii) genes targeted by SOX2 TF partners. This is because SOX2 forms dimers with an important share of TFs that are either selected with, or regulating a significant number of genes selected with, domestication (Table 4). This proposed central role of SOX2 in domestication events might be seen as supporting (a refined version of) the NC hypothesis of domestication. Indeed, *SOX2* in vertebrate embryos is abundantly expressed by a subpopulation of multipotent stem cells in the neural plate border, the embryonic structure from which the NC emerges. Besides NC cells, such neural plate border stem cells generate 3 other lineages: (i) neural progenitor cells giving rise to neurons and glial cells of the brain; (ii) the craniofacial placodes forming the sensory organs supporting the visual, auditory, and olfactory functions; and (iii) the cranial epidermis (Pla and Monsoro-Burq 2018; Thawani and Groves 2020; Milet and Monsoro-Burq 2012). These three lineages, along with the NC cell lineage, appear to be relevant in the context of domestication: to ensure a proper head formation, the fate of neural plate border stem cells is finely tuned by the expression of Sox2 and a few other TFs (Kimura-Yoshida et al. 2015; Thier et al. 2019). More specifically, SOX2 in neural plate border stem cells acts as a rheostat TF controlling the balance between two distinct cell fates: neural progenitors vs NC cells (Mandalos et al. 2014; Mandalos and Remboutsika 2017; Roellig et al. 2017; Mandalos et al. 2023). Aside from its role in neural plate border stem cells, SOX2 is necessary for the physiological development of the hypothalamic-pituitary-adrenal axis in mammals (Kelberman et al. 2006; Jayakody et al. 2012). As noted, these are the main endocrine organs impacted by domestication events (Belyaev 1979; Künzl and Sachser 1999; Herbeck et al. 2021).

The possibility that, as noted, the TFs involved in domestication might significantly target pathways important for the development of the brain and the craniofacial region is interesting, in view of the skull, brain, and even cognitive changes brought about by domestication (Kruska 2005; Trut et al. 2009a; Zeder 2012; Wilkins et al. 2014b; Hecht et al. 2023), but also if one considers the face, brain, and cognitive differences between humans and other extant and extinct hominid species, with our species claimed to have gone through a self-domestication process, as noted in the introduction (here *self* means that we might have followed an evolutionary path similar to domesticated mammals in absence of a domesticator agent) (see Hare 2017; Wrangham 2019 for details). This significant involvement of domestication-associated TFs in the development of the brain and the craniofacial region is reinforced by the specific roles performed by some of our core TFs, particularly, NR3C1 and NR3C2, as glucocorticoid activity is key for regulating basic aspects of brain development and function, including dendritic spine activity (Maggi et al. 2013; Saaltink and Vreugdenhil 2014; Uchoa et al. 2014; Koning et al. 2019). Also of interest, the core domestication-associated TFs we have identified in the paper target sets of genes that are likewise related to brain development (“axon guidance” and “BDNF signaling” pathways), but also to immunity (“Interleukin-2 signaling pathway”). Adaptive immunity is known to be impacted by domestication (Chen et al. 2017; Zheng et al. 2020; Suzuki and Okanoya 2021). Interestingly, it is known to also play a key role in brain development via the gut-brain axis (Zengeler and Lukens 2021). Furthermore, we found that the targets of our core domestication-associated TFs also share an enrichment in genes involved in the VEGFA/VEGFR2 pathway. VEGF signaling in the NC participates in the process of vessel formation in the cranial region, a fundamental step for the increase of the telencephalon in gnathostomes (Etchevers et al. 1999; Etchevers et al. 2001). Of note, besides angiogenesis, the VEGFA/VEGFR2 pathway is involved in axon guidance (Luck et al. 2019) and neuronal differentiation (Mackenzie and Ruhrberg 2012).

Indirectly, our findings can help clarify the mechanisms accounting for some other domestication features. In this regard, we find of interest the shared enrichment in the “regulation of extra-cellular matrix” pathway, which can be related to the morphological changes found in most domesticates in particular, changes in the conjunctive tissue (which could account for the floppy ears and tail-form modifications typically found in domesticated animals). Additionally, the enrichment in the adipogenesis pathway could explain the links between domestication and diet (Axelsson et al. 2013; Jin et al. 2020). Finally, we also find it relevant that some of the genes and pathways we have highlighted in the paper (particularly, the Wnt signaling pathway) are involved in the NC-mediated development of the adrenal cortex, as suggested by the fact that, in chicks, Wnt antagonists are expressed in migrating cephalic and truncal NC cells, and, ultimately, in NC derivatives (Duprez et al. 1999). Because the adrenal glands are one key target of domestication processes, our findings can be seen as reinforcing the suggested connection between the physiological triggers of domestication events, NC activity, and domestication-associated TF activity.

We conclude that, although domestication usually results in changes in many different body parts, alterations in TF activity might mostly impact the development and function of the brain and craniofacial region. This finding can be of particular interest for future studies aimed at understanding the behavioral and cognitive consequences of domestication (and self-domestication). That said, further research is needed to identify the molecular and physiological processes that are specifically contributed by the TFs we have highlighted in the paper and to clarify whether they play a direct role in domestication events. More importantly, this additional research should help properly test the possibility, implicit in our approach, that the disparate developmental genomic mechanisms underlying domestication are orchestrated by a core set of TFs.

### Supplementary information

ESM 1 Supplemental data file 1. Candidate genes for mammal domestication (XLSX 160 kb)

ESM 2 Supplemental data file 2. GO analyses of TFs selected in domesticated mammals. (XLSX 38 kb)

ESM 3 Supplemental data file 3. GO analyses of TFs regulating genes selected in domesticated mammals. (XLSX 305 kb)

ESM 4 Supplemental data file 4. GO analyses of TFs KLF4, MITF, NR3C1, and SOX2. (XLSX 2673 kb)

ESM 5 Supplemental data file 5. Functional partners of TFs KLF4, MITF, NR3C1, NR3C2, and SOX2 among the TFs selected in domesticated mammals and the TFs regulating genes selected in domesticated mammals. (XLSX 49 kb)

## Data Availability

The data extracted from databanks or generated by bioinformatics analyses are presented as tables or figures in the manuscript main document and in the supplementary materials.
